# Mammary myofibroblastoma of the male breast: a case report and literature review

**DOI:** 10.1308/rcsann.2024.0076

**Published:** 2024-10-22

**Authors:** R Elayyan, M Rizk, C Shah, R Price, N Garg

**Affiliations:** King’s College Hospital NHS Foundation Trust, UK

**Keywords:** Breast, Myofibroblastoma, Breast pathology, Breast tumours, Male breast

## Abstract

Mammary myofibroblastoma (MFB) is a rare benign spindle cell tumour predominantly affecting males, but also observed in postmenopausal females. Its diagnosis remains challenging owing to overlapping histological features with malignant lesions and limited tissue sampling in core biopsies. We present a case of incidentally discovered mammary MFB in a 63-year-old man and review its clinical, radiological and histopathological characteristics. The patient, who had a history of distal pancreatectomy and splenectomy, presented with an incidental left anterior chest wall nodule discovered on computed tomography scan. Clinical examination revealed a benign left retroareolar lump, confirmed by breast ultrasound and mammography. Ultrasound-guided core biopsy demonstrated characteristic spindle cells, prompting immunohistochemical staining confirming the diagnosis of MFB. The lesion was surgically excised with clear margins. Mammary MFB is commonly seen in postmenopausal women and older men, presenting as painless, mobile breast lumps. Imaging findings are nonspecific, resembling fibroadenomas or fat necrosis. Histologically, MFB lacks mammary ducts or lobules and displays characteristic spindle cells with collagenous stroma. Immunohistochemistry aids in differentiating it from other spindle cell tumours. Surgical excision is curative, with no reported cases with recurrence. Mammary MFB should be considered in the differential diagnosis of breast masses in males and postmenopausal women. Despite the challenges in diagnosis, its benign nature and favourable prognosis warrant timely recognition and appropriate management through surgical excision. Further research is needed to establish clear management guidelines and explore its underlying pathogenesis.

## Background

Myofibroblastoma (MFB) is a rare benign spindle cell tumour of the breast, historically reported with a male predominance.^1^ However, with the introduction of breast screening programmes, the gender distribution has become more equal. In females, it is more commonly observed in postmenopausal women.^2^

Around 90 case reports of mammary MFB have been documented to date, following its initial description as a separate entity in 1987 by Wargotz *et al*.^3^ Despite being rare, extramammary cases were reported, mainly along the embryonic milk line, which runs from the axilla to the inguinal area.^4^ Moreover, cases of extramammary MFB occurring in locations beyond this milk line have been reported.^5,6^ It has been reported to be associated with prostate disease treatment, gynaecomastia, radiation therapy, at the site of surgical scars, and in male-to-female transgender patients undergoing feminising hormone therapy.^4,7–10^ The most common presentation is a painless lump. These lumps may exhibit gradual and consistent growth over months to years.^11^ However, MFB may be discovered incidentally. Research questions regarding the prevalence, clinical presentation and optimal management of MFB remain underexplored. This case report aims to contribute to the existing literature by addressing these gaps. The standard approach for diagnosis is by triple assessment, which encompasses evaluating the patient clinically, utilising suitable imaging studies and conducting a core needle biopsy.^12^ Methods for the literature review involved searching databases such as PubMed and Google Scholar using keywords like ‘myofibroblastoma’, ‘breast tumour’ and ‘benign spindle cell tumour’. Relevant articles were selected based on their relevance and contribution to the understanding of mammary MFB (Table 1).

**Table 1:** Literature review

Article/year of publication	Age (years)	Gender	Presentation	Size (mm)	Management	Follow-up duration (months)
Abeysekara *et al*, 2008^13^	65	Male	Breast enlargement	150	Mastectomy	60
Mele *et al*, 2011^14^	65	Male	Breast lump	100	Surgical excision	–
Kataria *et al*, 2012^11^	73	Male	Breast lump	160	Surgical excision	–
Yildiz *et al*, 2016^15^	80	Male	Breast lump	30	Surgical excision	
Fügen *et al*, 2016^16^	35	Female	Breast lump	30	Excisional biopsy	15
Metry *et al*, 2016^17^	82	Male	Breast lump	15	Surgical excision	–
Boudaouara *et al*, 2017^18^	43	Female	Incidental radiological findings + palpable lump	20	Excisional biopsy	7
Jing *et al*, 2017^19^	42	Female	Breast lump	15	Surgical excision	20
Allahverdi and Allahverdi, 2017^20^	61	Male	Breast lump	80	Surgical excision	36
Comer *et al*, 2017^21^	73	Male	Incidental radiological finding – CT scan	9	Wide local excision	–
Shintaku *et al*, 2017^22^	56	Female	Painless induration	23	Surgical excision	–
Rochlis and Germaine, 2017^23^	50	Male	Incidental radiological finding – CT scan	8	Surgical excision	–
Khatib *et al*, 2018^3^	55	Female	Incidental radiological finding – screening mammogram	20	Surgical excision	–
Viswanathan *et al*, 2018^24^	74	Male	Bilateral breast lumps	Bilateral multiple, largest 17	Clinical observation	96
O'Bryan *et al*, 2018^8^	76	Transgender – male-to-female	Incidental –mammogram	18	Surgical excision	–
Shanmugasiva *et al*, 2018^25^	80	Male	Breast enlargement	36	Wide local excision	–
Yilmaz *et al*, 2018^26^	53	Male	Breast lump	–	Nipple-sparing mastectomy	–
Al Shammri *et al*, 2018^27^	70	Female	Breast lump	110	Surgical excision	6
Akrami *et al*, 2019^28^	65	Male	Breast lump	40	Modified radical mastectomy	60
Ross *et al*, 2019^29^	36	Female	Axillary fullness – incidental on ultrasound	16	Excisional biopsy	–
Fakim *et al*, 2019^30^	52	Female	Incidental radiological finding – CT scan	7	Vacuum-assisted excision	–
Jung *et al*, 2020^1^	52, 61	Both females	Incidental radiological finding – CT scan	16, 15	Surgical excision	52, 72
Venturelli *et al*, 2020^31^	65, 76	Both males	Incidental radiological finding – CT scan	31, 12	Mastectomy and axillary dissection mastectomy and sentinel lymph node biopsy	–
Scardina *et al*, 2021^32^	80, 59	Male, Female	Breast lump	36, 20	Surgical excision	–
Strait *et al*, 2021^33^	61	Female	Incidental –excision during surgery	15	Surgical excision	7
Bağlan *et al*, 2021^34^	62	Male	Incidental radiological finding – CT scan	3.8	Surgical excision	6
Wordekemper *et al*, 2022^35^	50’s	Male	Incidental radiological finding – CT scan	28	Surgical excision	–
Koufopoulos *et al*, 2022^36^	37	Female	Breast lump	32	Surgical excision	55
Inaishi *et al*, 2022^37^	74	Female	Breast lump	22	Surgical excision	–
Mečiarová and Pohlodek, 2023^38^	47	Female	Breast lump	10	Surgical excision	–
Jumana A Fatani *et al*, 2023^39^	76	Male	Breast lump	55	Surgical excision	–
Ferreira *et al*, 2023^40^	55	Male	Incidental radiological finding – CT scan	25	Surgical excision	7
Anna *et al*, 2023^41^	61	Male	Breast lump	25	Surgical excision	12

Pathologists often face challenges when distinguishing between benign and malignant lesions of MFB because of the rarity of these tumours and the overlap in histological features between benign and malignant forms. Furthermore, the limited tissue sample obtained by core biopsy may not provide enough information for a definitive diagnosis; therefore, an excision is often necessary for a more comprehensive evaluation, and this decision is based on clinical, radiological and pathological findings.

We report a case of incidentally found mammary MFB in a 63-year-old man, confirmed by immunohistochemical staining, which was surgically excised, followed by a review of the literature.

## Case history

**Figure 1:**
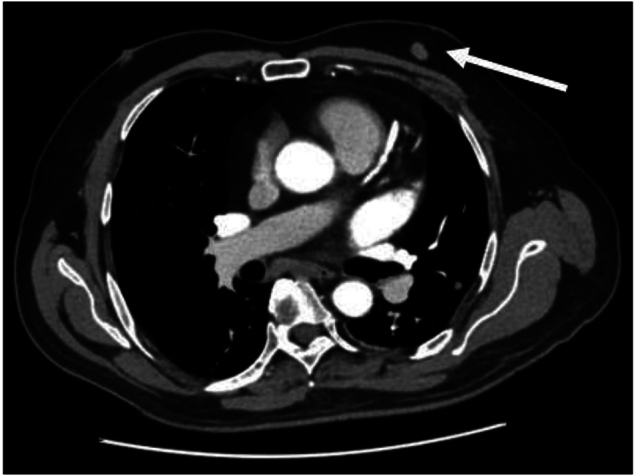
Computed tomography scan showing a 12mm left anterior chest wall lesion

A 63-year-old man was referred to our breast unit with an incidental computed tomography (CT) scan finding of a left anterior chest wall nodule, which had become enlarged from 8mm to 12mm in comparison with a previous CT scan done 7 years previously (Figure 1). He had a history of distal pancreatectomy and splenectomy for pancreatic neuroendocrine tumour 12 years ago. He was known to have insulin-dependent diabetes mellitus, was a non-smoker and had no family history of breast cancer.

**Figure 2:**
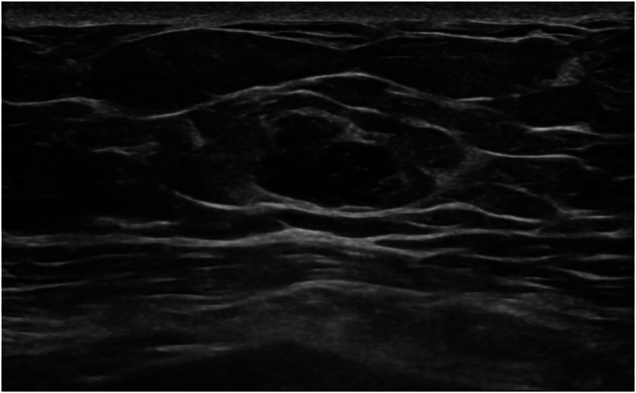
Breast ultrasound showing a 13mm ill-defined hypoechoic mass

**Figure 3:**
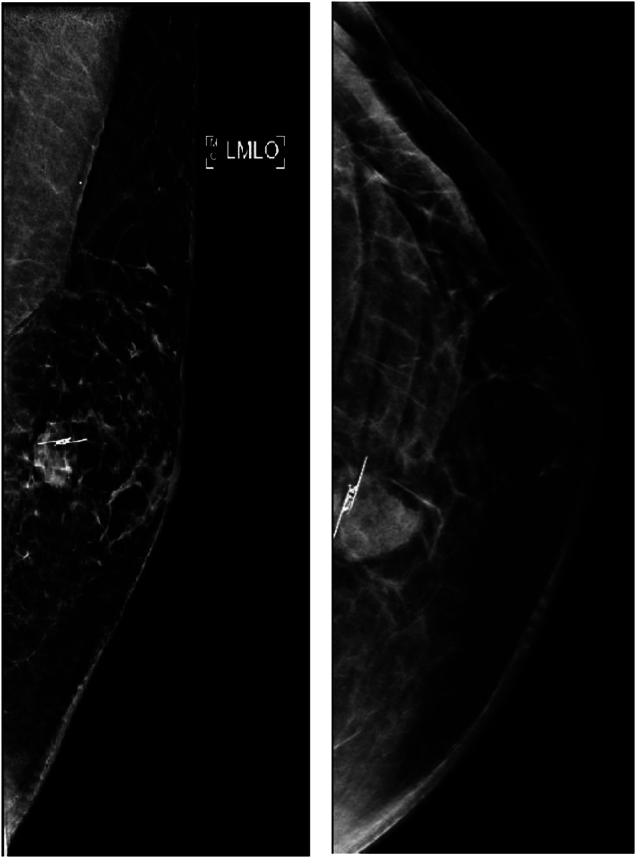
Left breast mediolateral oblique and craniocaudal tomosynthesis mammography views showing the placement of the SAVI SCOUT marker in the circumscribed tumour in the upper left breast

The patient presented with intermittent breast pain, and examination revealed a 15mm clinically benign left retroareola lump with no regional lymphadenopathy. The imaging by breast ultrasound scan and mammography showed a 13mm ill-defined mass in the left breast’s upper inner quadrant. No other solid or cystic lesion was seen within the left breast (Figures 2 and 3).

**Figure 4:**
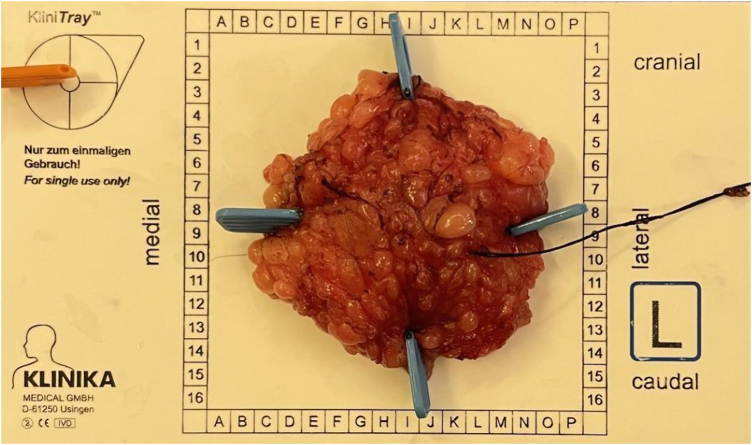
Surgical specimen oriented using specimen orientation kit ‘Klin tray’ and sutures

**Figure 5:**
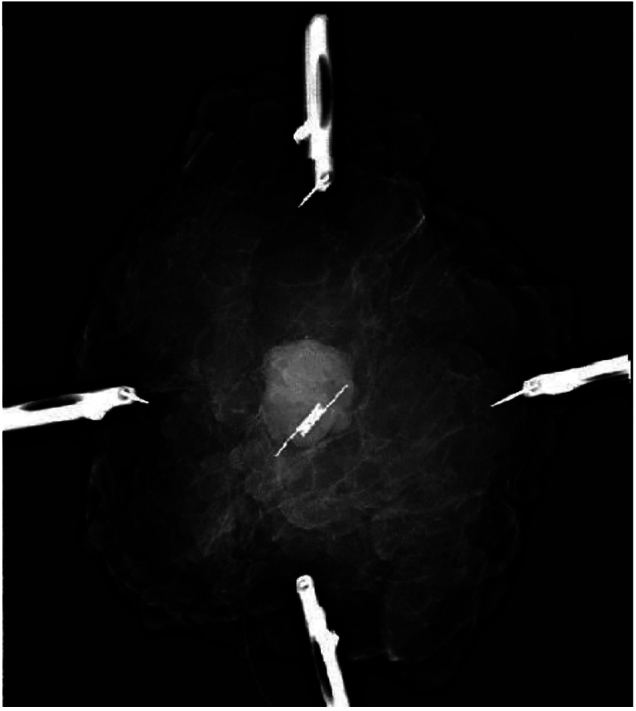
Intraoperative x-ray of the excised specimen showing the SAVI SCOUT located centrally within the lesion

Ultrasound-guided core biopsy showed part of a lesion composed of variably cellular areas of spindle-shaped cells arranged in short fascicles and a haphazard pattern. These cells contained a small amount of pale eosinophilic cytoplasm and elongated nuclei with blunt or tapered ends. In places, bands of hyalinised collagen bundles were present. Cytological atypia and necrosis were not a feature. Almost no mitoses and no breast ducts and lobules were seen.

Immunohistochemical stains performed showed that the spindle cells were positive for CD34, desmin, CD10 (with focal positivity) and CD56 (focal aberrant staining). The lesional cells were negative for cytokeratin markers (cytokeratin AE1/3 and epithelial membrane antigen (EMA)), smooth muscle actin (SMA), B-catenin and chromogranin. The proliferation index (Ki67) was 2–3%. Morphologic appearances together with immunophenotype were most suggestive of MFB. Excision of the specimen was recommended.

A multidisciplinary team meeting (MDT) recommended image-guided excision of the lump. SAVI SCOUT (nonradioactive surgical radar localisation technology) was inserted under ultrasound guidance. Digital breast tomosynthesis post insertion showed the SAVI SCOUT (Figure 3). Surgical excision was performed and the specimen X-rayed and oriented as per local protocol (Figures 4 and 5).

The postoperative recovery period was uneventful.

**Figure 6:**
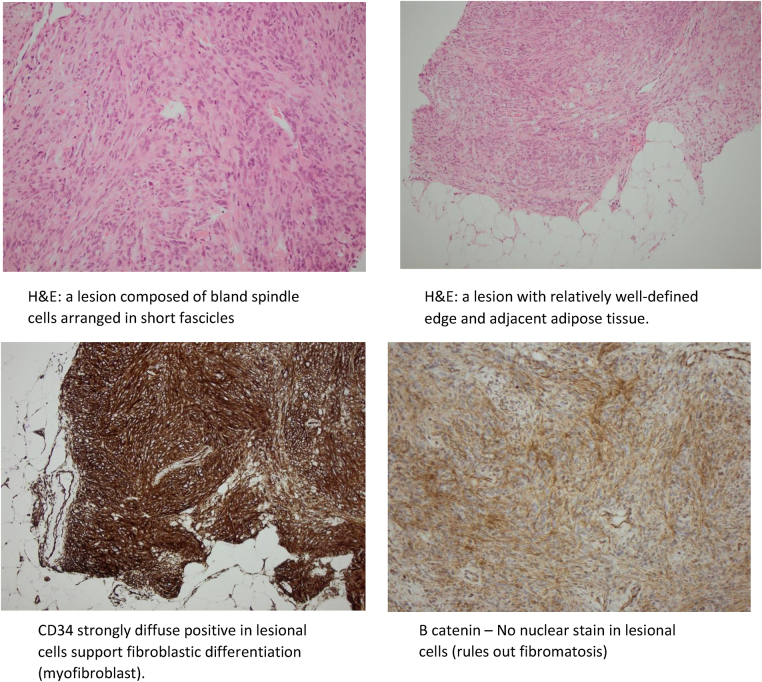

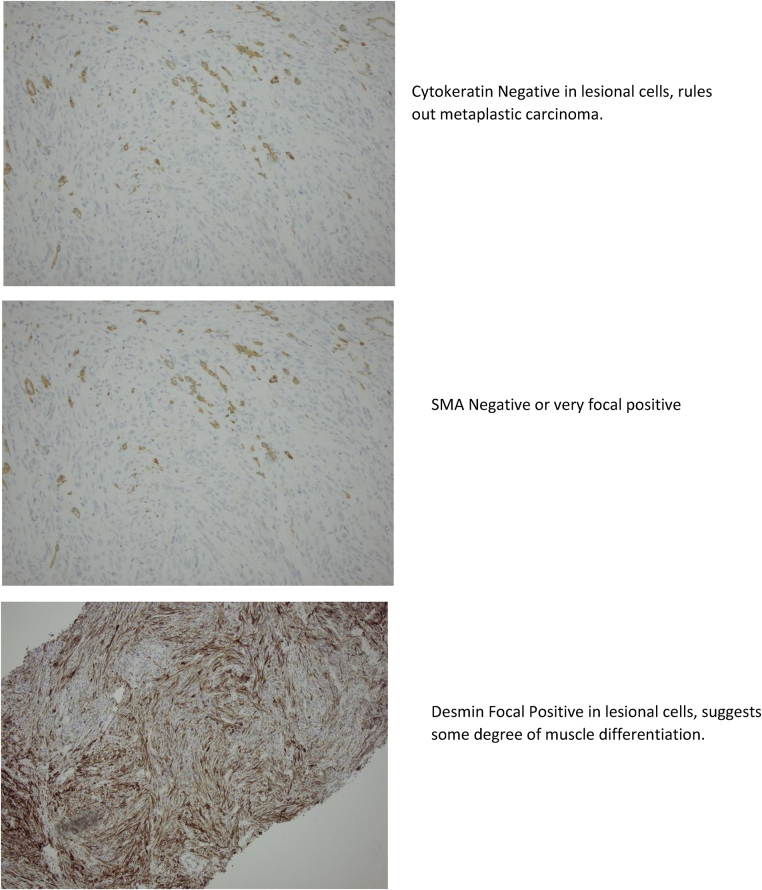
(a) Lesion composed of bland spindle cells arranged in short fascicles (haematoxylin and eosin [H&E] stain). (b) Lesion with relatively well-defined edge and adjacent adipose tissue (H&E). (c) CD34 strongly diffuse positive in lesional cells support fibroblastic differentiation (myofibroblast). (d) No nuclear stain in lesional cells (rules out fibromatosis) (B-catenin). (e) Cytokeratin negative in lesional cells, rules out metaplastic carcinoma. (f) SMA negative or very focal positive. (g) Desmin focal positive in lesional cells, suggests some degree of muscle differentiation

Postoperative processing of the specimen showed a white soft circumscribed lesion where all margins were clear for more than 2mm. Sections from the breast excision specimen showed a relatively well-circumscribed but non-capsulated spindle cell lesion. The spindle cells were arranged in short fascicles, occasionally showing a storiform pattern. Interspersed adipose tissue and collagen bundles were noted occasionally. A focal area of multinucleated cells was also evident. No cytological atypia or necrosis were present and mitoses were inconspicuous. Few benign ducts and lobules were present in the surrounding tissue. Immunostains performed showed that the spindle cells were positive for H-caldesmon, CD34, desmin and CD10. SMA is very focally positive. The spindle cells were negative for S100p, AE1/3 and EMA. Proliferation was low. The appearances were of MFB with no atypical features. The excision was complete with at least 2mm clearance to the closest margin (Figure 6).

The MDT consensus was to reassure and discharge the patient because there was no need for any further adjuvant treatment.

## Discussion

Myofibroblastoma is a rare benign tumour originating from the mesenchymal tissue and exhibits differentiation towards myofibroblastic cells.^32^ In 1981, Toker *et al* initially reported four cases of benign stromal tumours in the breast, which exhibited morphological characteristics similar to spindle cell lipomas of soft tissue and were termed benign spindle cell tumours of the breast. The specific designation of MFB for this distinct entity was introduced later in 1987 by Wargotz *et al*.^17,20,25,42^

The majority of mammary MFB cases occur between the ages of 40 and 87 years. Postmenopausal women and older men are typically affected.^43,44^ There is evidence of equal sex distribution,^45^ possibly because of increased screening mammograms.^3^ There is no proof to suggest that MFB is associated with any particular ethnicity, gender, medical condition, drug or hormone supplements.^14^ The coexistence of gynaecomastia and MFB is also very rare.^17,46,47^

The typical presentation is a unilateral, mobile, painless lump, whereas bilaterality and multicentricity are rare.^48^ MFB does not show any specific pattern on imaging. On an ultrasound scan, it is a uniformly hypoechoic, well-circumscribed solid round to oval mass with a variable posterior attenuation, and on a mammogram usually shows a non-calcified mass that is round or oval in shape and well circumscribed.^2,49^ It has benign sonographic features and resembles fibroadenoma in women and fat necrosis, angiolipoma and pseudo-angiomatous stromal hyperplasia in men.^26,50^ A broader spectrum of differential diagnoses among females often complicates the quick and precise identification of this uncommon condition.^51^

It is challenging to establish the diagnosis of this neoplasm through cytology and needle biopsy. However, it is relatively straightforward on a resected specimen.^52^ Mammary MFB is a benign growth originating from mesenchymal cells and linked to the deletion of chromosome 13q14, resembling spindle cell lipoma and cellular angiofibroma.^46^ Histologically, MFB consists of spindle-shaped cells arranged in short intersecting fascicles, with interruptions by keloid-like eosinophilic collagen bands. Notably, mammary ducts or lobules are not present. On a macroscopic level, the cut surface displays a distinctly defined, pale pink or tan rounded mass.^53,54^ Mitoses are limited; with fewer than 2 per 10 high-power fields. When an in situ component is present, it suggests a likelihood of carcinoma diagnosis.^54^

When there is a suspicion of MFB based on microscopic examination, conducting immunohistochemical staining using antibody panels to differentiate MFB from other lesions is crucial. Immunohistochemical analysis reveals that MFB displays positivity for CD34, CD10, vimentin, CD99, oestrogen receptors, progesterone receptors and BCL-2 protein. It also shows varied positivity for androgen receptors, SMA, H-caldesmon and desmin. On the other hand, MFB tests negative for pan-cytokeratin, CD117 (C-kit), EMA, HMB-45 and S100. These findings correspond with the fibroblastic and myofibroblastic characteristics of the tumour cells. Other spindle cell tumours like pseudoangiomatous stromal hyperplasia, spindle cell lipoma, nodular fasciitis, solitary fibrous tumour, leiomyoma, fibromatosis and metaplastic spindle cell carcinoma are among the differential diagnoses for breast MFB.^55^

MFBs can be classified into five distinct microscopical types: epithelioid, collagenised, classical, cellular and infiltrative. The typical histological types lack the lobules and the mammary ducts, and the surrounding breast parenchyma may create a pseudocapsule.^56^ The expression of oestrogen, progesterone and androgen receptors indicates that mammary MFB may be related to steroid hormones and their receptors.^57^

MFB can be managed by surgical excision primarily to relieve symptoms.^48,58,59^ Although surgery is not obligatory because of the benign nature of the lesion, uncertainty about the long-term implications of an unresected MFB arises from previous case reports in which surgical removal was the only standard to approach myofibroblastoma.^12^

MFB is not known to exhibit local recurrence, and surgical excision is thought to be curative. Despite no evidence to suggest malignant potential, some researchers recommend 24-month follow-up for this rare phenomenon to increase knowledge and understanding.^12^

## Conclusion

Mammary MFB is a rare breast tumour that should be considered a differential diagnosis in postmenopausal women and older men. A precise diagnosis can be achieved by clinical, radiological and pathological findings, and there is no evidence to suggest a risk of malignant transformation, metastasis or recurrence. It is a relatively new diagnosis with limited epidemiological information and clear management guidelines.
